# Emergence of a *Salmonella* Rissen ST469 clinical isolate carrying *bla*_NDM-13_ in China

**DOI:** 10.3389/fcimb.2022.936649

**Published:** 2022-08-08

**Authors:** Yulan Huang, Xiaobo Ma, Shihan Zeng, Liang Fu, Heping Xu, Xiaoyan Li

**Affiliations:** ^1^ Department of Clinical Laboratory, Fifth Affiliated Hospital, Southern Medical University, Guangzhou, China; ^2^ Department of Clinical Laboratory, the First Affiliated Hospital of Xiamen University (Xiamen Key Laboratory of Genetic Testing), School of medicine, Xiamen University, Xiamen, China; ^3^ School of Public Health, Xiamen University, Xiamen, China

**Keywords:** *bla*_NDM-13_, *Salmonella* Rissen, ST469, IS*Aba125*, IS*1294*

## Abstract

New Delhi metallo-β-lactamase-13 (NDM-13) is an NDM variant that was first identified in 2015 and has not been detected in *Salmonella* species prior to this study. Here we describe the first identification of a *Salmonella* Rissen strain SR33 carrying *bla*_NDM-13_. The aim of this study was to molecularly characterize SR33’s antimicrobial resistance and virulence features as well as investigate the genetic environment of *bla*_NDM-13_. The *Salmonella* Rissen SR33 strain was isolated from a patient with fever and diarrhea. SR33 belonged to ST469, and it was found to be multidrug-resistant (MDR) and to carry many virulence genes. Phylogenetic analysis showed that SR33 shared a close relationship with most of the Chinese *S*. Rissen ST469 strains. *bla*_NDM-13_ was located in a transmissible IncI1 plasmid pNDM13-SR33. Sequence analysis of *bla*_NDM-13_-positive genomes downloaded from GenBank revealed that a genetic context (ΔIS*Aba125*-*bla*_NDM-13_-*ble*_MBL_-trpF) and a hybrid promoter (consisting of −35 sequences provided by IS*Aba125* and −10 sequences) were conserved. IS*Aba125* was truncated by IS*1294* in three plasmids carrying *bla*_NDM-13_, including pNDM13-SR33. To our knowledge, this is the first report of *bla*_NDM-13_ carried by *Salmonella*. The emergence of *bla*_NDM-13_ in a clinical MDR *S*. Rissen ST469 strain highlights the critical need for monitoring and controlling the dissemination of *bla*_NDM-13_. *bla*_NDM-13_ carried by a transmissible IncI1 plasmid may result in an increased risk of *bla*_NDM-13_ transmission. IS*1294* may be involved in the movement of *bla*_NDM-13_.

## Introduction

Carbapenems have been used for decades to treat severe gram-negative bacterial infections, particularly in resistant and multidrug-resistant (MDR) infections (Hansen, 2021). According to the World Health Organization’s Global Priority List, carbapenem-resistant Enterobacteriaceae (CRE) pose a growing threat to public health worldwide (Tacconelli et al., 2018). New Delhi metallo-β-lactamase (NDM) is a subclass B1 metallo-β-lactamase that is capable of hydrolyzing almost all β-lactams including carbapenems (Yong et al., 2009; Nordmann et al., 2011). Worse still, clinically available β-lactamase inhibitors are ineffective in preventing carbapenem hydrolysis by NDM enzymes (Wu et al., 2019). NDM-positive strains are usually resistant to most of antimicrobial agents, due to coexistence of other resistance mechanisms (Nordmann et al., 2011), leading to a variety of infections that are associated with high mortality (Guducuoglu et al., 2017). Since NDM-1 was first identified in clinical isolates in India in 2008 (Yong et al., 2009), 31 variants have been reported worldwide, representing a significant challenge for public health and clinical management (Moellering, 2010; Dortet et al., 2014; Li et al., 2021). Of these, NDM-13 is a variant that has two amino acid substitutions (D95N and M154L) compared with NDM-1, resulting in the increased hydrolytic activity against cefotaxime (Shrestha et al., 2015). NDM-13 has been detected in five *Escherichia coli* strains obtained from Nepal (n = 1) (Shrestha et al., 2015), China (n = 3) (Lv et al., 2016), and Korea (n = 1) (Kim et al., 2019). Here we aim to characterize a *bla*_NDM-13_-positive *Salmonella* Rissen strain SR33 isolated in China. To our knowledge, this is the first report of *bla*_NDM-13_ detected in *Salmonella*.

## Materials and methods

### Bacterial strain

Strain SR33 was isolated from a fecal sample of an old patient. This patient was hospitalized due to occasional fever and diarrhea. During hospitalization, cefixime was ineffective against this infection, but it improved after treatment with levofloxacin. SR33 was identified by the VITEK-2 COMPACT automatic microbial identification system (bioMérieux, Marcy-l’Étoile, France), and its serotype was confirmed by slide agglutination technique (Kauffmann-White-Le Minor scheme) (Grimont and Weill, 2007)

### Antimicrobial susceptibility testing

The minimum inhibitory concentrations (MICs) for imipenem, ertapenem, ceftazidime, ceftriaxone, cefepime, amoxicillin/clavulanic acid, piperacillin/tazobactam, trimethoprim/sulfamethoxazole, levofloxacin, ampicillin, tetracycline, ciprofloxacin, chloramphenicol, and azithromycin were determined by broth microdilution following the CLSI guidelines, and MIC results were interpreted according to the CLSI breakpoints (Wayne, 2021).

### Whole-genome sequencing and bioinformatics analysis

The genomic DNA of SR33 was extracted by the bacterial genomic DNA extraction kit (Tiangen, Beijing, China) and sequenced on an Oxford Nanopore platform (Novogene, Tianjin, China). Sequence reads were assembled by Unicycler 0.4.8 (Wick et al., 2017) and annotated by Prokka 1.14.5 (Seemann,, 2014). The serotype was further confirmed by SISTR 1.1.1 (Yoshida et al., 2016), and the sequence type (ST) was determined using MLST 2.18.0 (Larsen et al., 2012). The distance matrix based on the core-genome single-nucleotide polymorphism (SNP) profiles of 37 Chinese *S*. Rissen ST469 isolates was generated using Parsnp and HarvestTools (Treangen et al., 2014). The phylogenetic tree was constructed by MEGA X (Kumar et al., 2018). Resistance genes and plasmid replicons were identified using Abricate (https://github.com/tseemann/abricate) with the ResFinder (Zankari et al., 2012) and PlasmidFinder (Carattoli et al., 2014) databases, respectively. The filtering criteria of antimicrobial resistance genes were >90% identity and >90% coverage. The virulence genes were analyzed by the database of Virulence Factors of Pathogenic Bacteria (VFDB) using BLASTn with a threshold of >70% identity and >70% coverage (Chen et al., 2016). The presence of *Salmonella* pathogenicity islands (SPIs) was explored by SPIFinder (https://cge.cbs.dtu.dk/services/SPIFinder/). Circular maps of plasmids were generated using the BLAST Ring Image Generator (BRIG) tool (Alikhan et al., 2011). Transposon and insertion sequence (IS) elements were scanned using the ISFinder database (Siguier et al., 2006). BLASTn (Altschul et al., 1990) was used to determine the identity of the genetic environment between NDM-13-positive sequences. The genetic environment was visualized by EasyFig (Sullivan et al., 2011).

### Plasmid conjugation experiments

Transferability of plasmid harboring *bla*_NDM-13_ was assessed by the conjugation experiment, using rifampin-resistant *E. coli* C600 as the recipient strain. Transconjugants were selected on Luria-Bertani agar plates containing rifampin (100 µg/ml) and imipenem (2 µg/ml). Transconjugants containing the *bla*_NDM-13_ gene were verified by PCR sequencing (forward primer sequence: ATGGAATTGCCCAATATTATGCAC and reverse primer sequence: TCAGCGCAGCTTGTCGGC). The antimicrobial susceptibility of the transconjugant was confirmed by the broth microdilution method.

### Nucleotide sequence accession number

The whole-genome sequence of SR33 has been submitted to the GenBank database with accession numbers CP092911–CP092914. The nucleotide sequence of plasmid pNDM13-SR33 has been deposited under accession number CP092912.

## Results

### Antimicrobial susceptibility testing and antimicrobial resistance genes

As shown in **Table 1**, SR33 was multidrug resistant to all tested β-lactams, trimethoprim/sulfamethoxazole, and tetracycline and was susceptible to quinolones (levofloxacin and ciprofloxacin), azithromycin, and chloramphenicol. In addition to *bla*_NDM-13_, SR33 carried genes that mediate resistance to β-lactams (*bla*_TEM-1_), bleomycin (*ble*_MBL_), streptomycin *(aadA1*, *aadA2*), chloramphenicol *(cmlA1*), trimethoprim (*dfrA12*), sulfonamide (*sul3*), and tetracycline [*tet(A)*]. The information of resistance genes detected in SR33 is listed in **Supplementary Table S1**.

**Table 1:** MIC values of antimicrobials for SR33 and its transconjugant.

	SR33		C600		SR33-C600	
Antimicrobials	MIC values (µg/mL)	Interpretation	MIC values (µg/mL)	Interpretation	MIC values (µg/mL)	Interpretation
Imipenem	≥16	R	≤1	S	≥16	R
Ertapenem	≥8	R	≤0.5	S	≥8	R
Ceftazidime	≥64	R	≤4	S	≥64	R
Ceftriaxone	≥64	R	≤1	S	≥64	R
Cefepime	16	R	≤2	S	16	R
Amoxicillin/clavulanic acid	≥32	R	≤4	S	≥32	R
Piperacillin/tazobactam	≥128	R	≤16	S	≥128	R
Trimethoprim/sulfamethoxazole	≥320	R	≤20	S	≤20	S
Levofloxacin	≤0.12	S	≤0.5	S	0.5	S
Ampicillin	≥32	R	≤8	S	≥32	R
Tetracycline	≥16	R	≤4	S	≤4	S
Ciprofloxacin	≤0.06	S	≤0.25	S	≤0.25	S
Chloramphenicol	≤8	S	≤8	S	≤8	S
Azithromycin	≤16	S	≤16	S	≤16	S

MIC, minimum inhibitory concentration; R, resistant; I, intermediate; S, sensitive.

Whole-genome sequencing (WGS) showed that *bla*_NDM-13_ and *ble*_MBL_ were located on an IncI1 plasmid designated as pNDM13-SR33, which is 88,258 bp in length with an average GC content of 50.37%. The other resistance genes were found on the chromosome. pNDM13-SR33 was successfully self-transferred into C600, and the transconjugant SR33-C600 was resistant to all tested β-lactams (**Table 1**).

### Characterization of the SR33 strain and phylogenetic analysis of Chinese *S.* Rissen ST469 isolates

**Figure 1:**
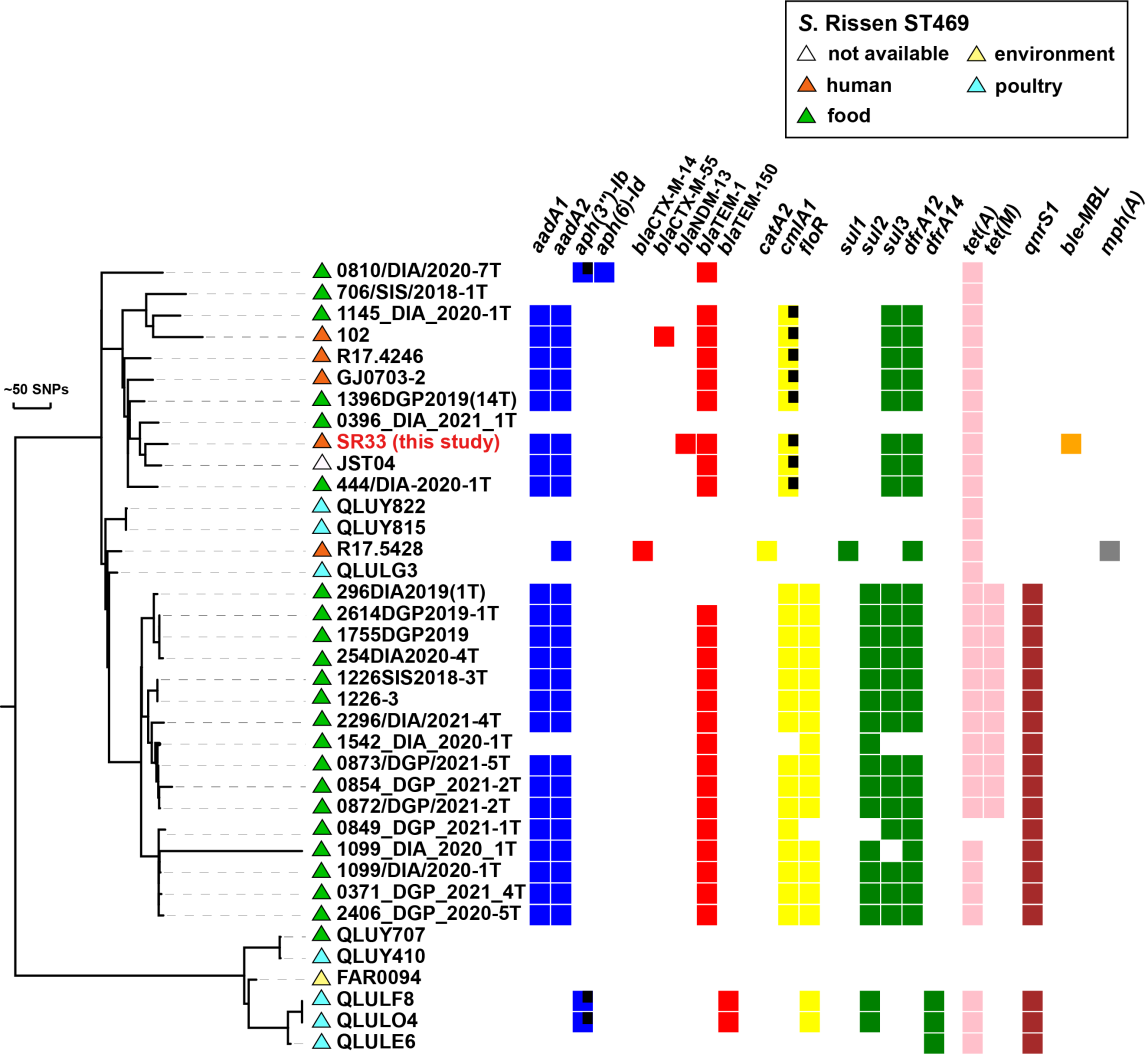
Phylogenetic distribution of antimicrobial resistance genes of SR33 of this study and other Chinese *S*. Rissen ST469 isolates. Antimicrobial resistant genes are represented by colored squares, genes with partial deletions are marked with black squares. The source of each isolate is shown with colored triangles. Bars represent unit distance of 50 SNPs.

The serotype and sequence type of SR33 were determined to be serovar Rissen and ST469. Phylogenetic analysis of SR33 with other 36 Chinese *S.* Rissen ST469 isolates (retrieved and downloaded from EnteroBase in February 2022, https://enterobase.warwick.ac.uk/species/index/senterica) revealed that SR33 differed from the other isolates by 41–418 SNPs (**Figure 1**). The information of these strains is listed in **Supplementary Table S2**. Besides, these strains were mainly isolated from food, poultry, and humans. Meanwhile, the majority of Chinese *S*. Rissen ST469 strains were MDR. The drug resistance profiles of these MDR strains were similar, and common drug resistance genes include *aadA1*, *aadA2*, *bla*_TEM-1_, *cmlA1*, *sul3*, *dfrA12*, and *tet(A)*. Since the common drug resistance genes in SR33 were located on chromosomes, and 29/37 Chinese *S.* Rissen ST469 isolates did not carry resistance plasmids, we speculated that the antimicrobial resistance genes were mainly located on the chromosomes of these closely related MDR strains.

### *Salmonella* pathogenicity islands and virulence-associated genes

According to SPIFinder, SR33 contained SPI-1 to SPI-5, SPI-8, and SPI-9. All VFDB-annotated genes are listed in **Table 2**. Based on the annotation of the VFDB database, SR33 harbored 124 virulence genes. The virulence genes are involved in adhesion systems, iron uptake, magnesium uptake, macrophage, flagella, type III secretion systems (T3SS), and serum resistance.

**Table 2:** Virulence-associated genes in SR33.

VF classes	Virulence factors	Genes
Fimbrial adherence determinants	Agf (thin aggregative fimbriae/curli)	*csg*ABCDEFG, *ste*ABC
	Lpf (long polar fimbriae)	*lpf*ABCE
	Type 1 fimbriae	*fim*CDFHI
Non-fimbrial adherence determinants	MisL	*mis*L
	SinH	*sin*H
Iron uptake	Enterobactin	*ent*ABCES, *fep*ABCDG
	Salmochelin	*iro*BCN
Magnesium uptake	Magnesium uptake/transporter	*mgt*BC
Macrophage inducible gene	Mig-14	*mig*-14
Motility	Flagella	*che*WY, *flg*GH, *fliA*GMP
Secretion system	T3SS (SPI-1 encoded)	*inv*ABCEFGHIJ, *org*ABC, *prg*HIJK, *sic*AP, *sip*D, *spa*OPQRS
	T3SS-1 translocated effectors	*avr*A, *sip*ABC/*ssp*ABC, *sop*ABDE2, *spt*P, *slr*P
	T3SS (SPI-2 encoded)	*ssa*CDEGHIJKLMNOPQRSTUV, *ssc*AB, *sse*ABCDE
	T3SS-2 translocated effectors	*pip*BB2, *sif*ABH, *sop*D2, *sse*FGJK1K2L, *spi*C/*ssa*B
Serum resistance	OmpA (Outer membrane protein A)	*omp*A
Others	Lipooligosaccharide	*gmh*A/*lpc*A
	Lipopolysaccharide	*gtr*AB

VF, virulence factors.

### Plasmid analysis of *bla*_NDM-13_-positive isolates

**Figure 2:**
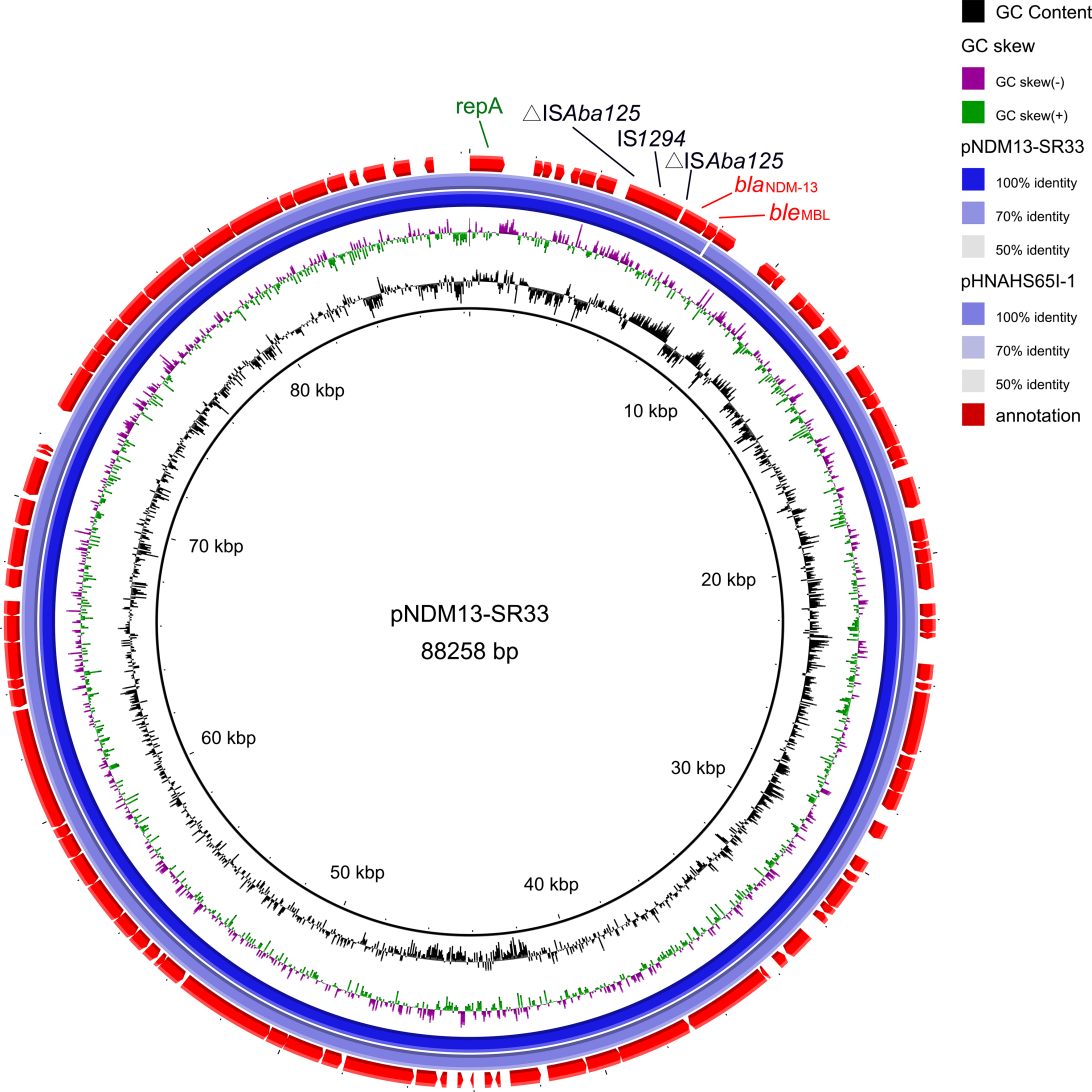
Genetic map of pNDM13-SR33 (no. CP092912) and pHNAHS65I-1 (no. MN219406). The repA, IS elements, resistance genes are annotated by green, black, red fonts, respectively.

NDM-13 has been identified in plasmids of three *E. coli* stains, including an IncX3 plasmid pNDM13-DC33(accession no. KX094555), an IncFIB plasmid pSECR18-0956 (accession no. MK157018), and an IncI1 plasmid pHNAHS65I-1 (accession no. MN219406). Of note, pNDM13-SR33 shared 99% coverage and 100% identity with an IncI1-*bla*_NDM-13_ plasmid pHNAHS65I (accession no. MN219406) of *E. coli* discovered in 2020 (**Figure 2**), which has a truncated *ble*_MBL_.

### Comparative analysis of the genetic environment of *bla*_NDM-13_

**Figure 3:**
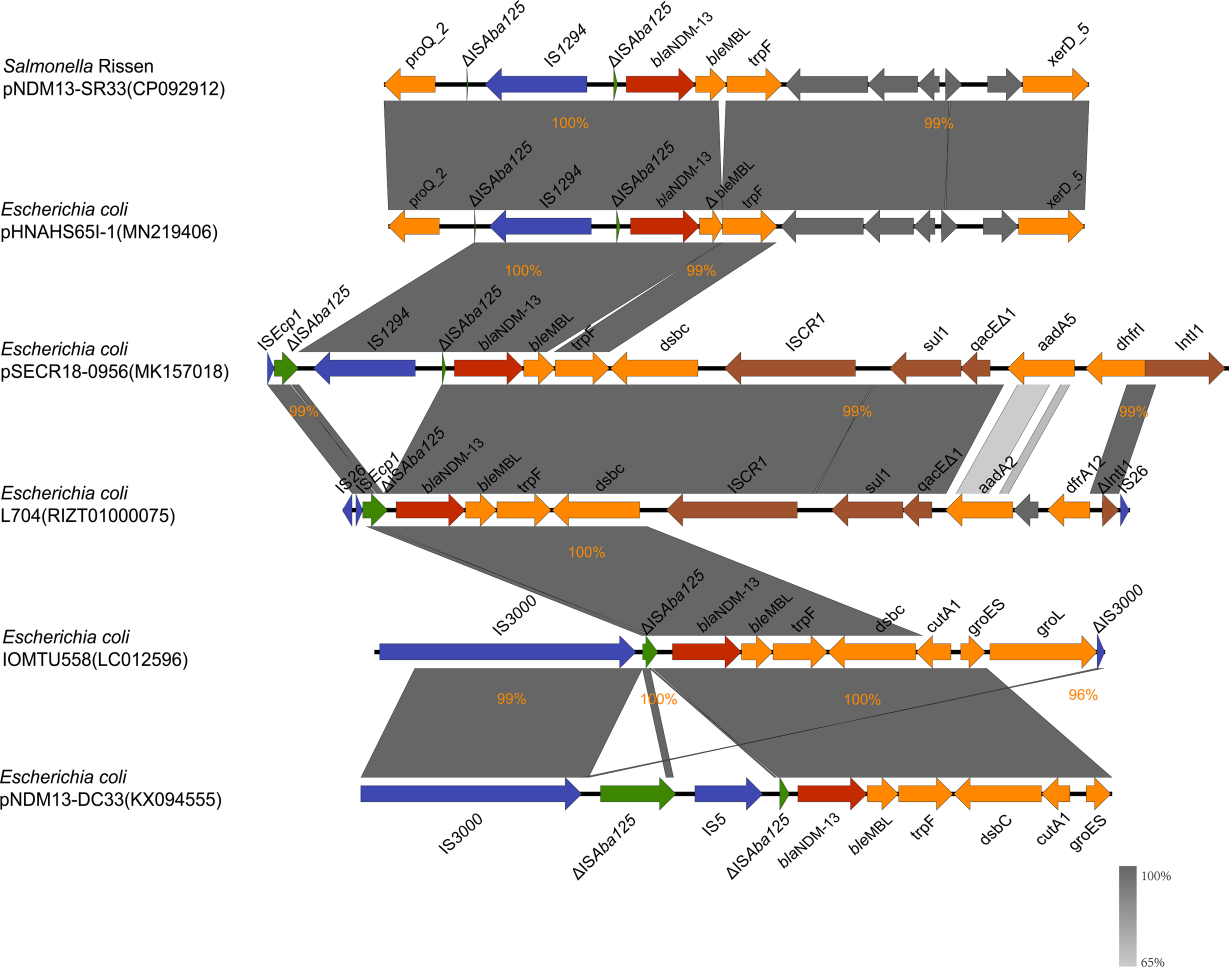
*bla*_NDM-13_ flanking sequence of pNDM13-SR33 (no. CP092912), pHNAHS65I-1 (no. MN219406), L704 (no. RIZT01000075), pSECR18-0956 (no. MK157018), IOMTU558 (no. LC012596), and pNDM13-DC33 (no. KX094555). The *bla*_NDM-13_ gene, IS*Aba125*, other insertion sequences, integron elements, and genes encoding hypothetical proteins are shown in red, green, blue, brown, and gray, respectively. The rest of genes are colored in orange. Δ; indicates a truncated gene or mobile element. The percentages signify the genetic identity between these sequences.

As shown in **Figure 3**, the *bla*_NDM-13_-producing strains shared a conserved genetic structure (ΔIS*Aba125*-*bla*_NDM-13_-*ble*_MBL_-trpF). The conserved region was found involved in various genetic contexts with different insertion sequences. The genetic context of *bla*_NDM-13_ in SR33 was highly similar to pHNAHS65I-1 (no. MN219406) with ΔIS*Aba125* truncated by the insertion of an IS*1294* upstream, which was also detected in pSECR18-0956 (no. MK157018). In L704 (no. RIZT01000075) and pSECR18-0956 (no. MK157018), the *bla*_NDM-13_ region was adjacent to an IS*CR1* complex class 1 integron (IS*CR1*-*sul1*-*qacEΔ1*-*IntI1*). The sequences of L704 and IOMTU558 (accession no. LC012596) were flanked by IS*26* and IS*3000*, respectively. In addition, a cluster (IS*3000*-ΔIS*Aba125*-*IS5*-ΔIS*Aba125*) was found upstream of *bla*_NDM-13_ in pNDM13-DC33 (no. KX094555). Moreover, a hybrid promoter (consisting of −35 sequences within the inverted repeat left of IS*Aba125* and −10 sequences) located upstream of *bla*_NDM-13_ was conservative in *bla*_NDM-13_-producing strains.

## Discussion

To date, New Delhi metallo-β-lactamase-13 (NDM-13) has been detected in five *E. coli* stains with different genetic backgrounds. Here, we report the emergence of an NDM-13-positive *Salmonella* strain SR33. The serotype of SR33 was determined to be serovar Rissen, which is regarded as one of the 20 most common serovars to cause human salmonellosis (European Food Safety Authority, E.C.f.D.P.a.C, 2017). SR33 was assigned to ST469, an MDR clone that has been reported in multiple countries (Campos et al., 2019).

SR33 was found to be MDR and to harbor nine resistance genes. These resistance genes were consistent with the phenotypes except for *cmlA1*. SR33 remained sensitive to chloramphenicol, which might be due to the fact that the *cmlA1* gene had a sequence deletion of 96 bp. Since SR33 was resistant to all β-lactams and susceptible to quinolones, it explains well why cefixime was ineffective against this infection and levofloxacin was effective.

Based on phylogenetic analysis, SR33 was closely related to the majority of the Chinese *S*. Rissen ST469 strains downloaded from EnteroBase. Since the available 37 Chinese *S*. Rissen ST469 isolates were mostly isolated from food, poultry, and humans, it is in agreement with the idea that *S*. Rissen infection occurs in humans as a zoonosis through food chain transmission (Xu et al., 2020). Therefore, it is possible that this patient had a foodborne infection. Another important finding is that most Chinese *S.* Rissen ST469 strains were MDR and shared similar drug resistance profiles. Since the antimicrobial resistance genes were mainly located on chromosomes, we should pay close attention to the vertical transmission of MDR *S*. Rissen ST469 strains. These observations emphasize the necessity of the surveillance of *S*. Rissen ST469 pathogens.

SPIs are gene clusters located on chromosomes and encode various virulence components (Foley et al., 2008). SR33 contained five important SPIs (SPI-1 to SPI-5) that are correlated with the pathogenesis of *Salmonella* (Cui et al., 2021) and additional two SPIs (SPI-8, SPI-9). Based on the annotation of the VFDB database, most of the virulence genes carried by SR33 are associated with flagella, type III secretion systems (T3SS), and adhesion systems, which have been demonstrated to play a variety of roles in the pathogenesis of *Salmonella* (Jajere, 2019). Of these, T3SS is regarded as the most important virulence factor of *Salmonella* (Lou et al., 2019). In general, MDR strain SR33 possessed important pathogenicity islands and many virulence-associated genes, which highlights the pathogenesis of SR33.

NDM-13 was first identified on the chromosome of *E. coli* IOMUT558 (ST101) from Nepal (Shrestha et al., 2015), and it was subsequently detected in four *E. coli* stains, namely, an IncFIB plasmid pSECR18-0956 of SECR18-0956 (ST8499) from Korea (Kim et al., 2019), an IncX3 plasmid pNDM13-DC33 carried by DC33 (ST5138) (Lv et al., 2016), an IncI1 plasmid pHNAHS65I-1 of AHS8C65RI, and L704 strain (the location of *bla*_NDM-13_ is unclear) from China. In our study, *bla*_NDM-13_ was found in a transmissible IncI1 plasmid pNDM13-SR33 of *S*. Rissen (ST469). The high coverage and identity between pNDM13-SR33 and pHNAHS65I-1 suggest that cross-species dissemination of *bla*_NDM-13_ plasmids had occurred. The *bla*_NDM_-carrying plasmids mostly belong to IncX3, IncFII, and IncC replicon types (Wu et al., 2019), indicating that the vector of NDM-13 may be different from the other variants. Previous studies showed that the IncI1 plasmids are often associated with clinically relevant strains (García-Fernández et al., 2008) and it is the major vehicle of extended spectrum β-lactamase (Carattoli et al., 2021). Thus, *bla*_NDM-13_ in SR33 carried by an IncI1 transmissible plasmid may result in an increased risk of *bla*_NDM-13_ transmission.

Comparative analysis of the *bla*_NDM-13_ genetic contents revealed that *bla*_NDM-13_ was bracketed by multi-insertional sequences. Of these, IS*Aba125* was conservative in *bla*_NDM-13_-positive isolates. It is consistent with the finding that IS*Aba125* (intact or truncated) upstream of *bla*_NDM_ is common in *bla*_NDM_ genetic contexts (Ahmad et al., 2018; Pérez-Vázquez et al., 2019; Das et al., 2019; Wu et al., 2019), implying a role in the transmission of *bla*_NDM_. IS*3000*, IS*26*, and IS*5* have also been reported to be associated with dissemination of NDM-encoding genes, while the role of IS*1294* is still unclear (Zhao et al., 2021; Acman et al., 2022). IS*1294* belongs to the IS*91* family, and previous reports demonstrated that the disruption of the IS*Ecp1* element by IS*1294* was linked to the promotion of *bla*_CMY-2_ (Sidjabat et al., 2014; Tagg et al., 2014) and *bla*_CTX-55_ (Pan et al., 2013; Hu et al., 2018) gene dissemination. In this study, ΔIS*Aba125* truncated by IS*1294* was found in three *bla*_NDM-13_-harboring plasmids including pNDM13-SR33. We thus suspected that IS*1294* may be involved in the mobilization and dissemination of *bla*_NDM-13_.

Expression of the *bla*_NDM-1_ gene is under the control of a hybrid promoter (consisting of −35 sequences within the inverted repeat left of IS*Aba125* and −10 sequences) located upstream of *bla*_NDM-1_ (Poirel et al., 2011). BLASTn analysis revealed that this hybrid promoter was also conservative in *bla*_NDM-13_-producing strains. This finding further supports that *bla*_NDM-13_ is derived from *bla*_NDM-1_ (Lv et al., 2016; Wu et al., 2019).

## Conclusion

To the best of our knowledge, this study first reports an NDM-13-producing *Salmonella* isolate. The emergence of *bla*_NDM-13_ in a clinical MDR *Salmonella* Rissen ST469 strain poses a significant threat to public health. Most of the *S*. Rissen ST469 strains isolated from China were MDR, which highlights the importance of the surveillance for *S*. Rissen ST469. The *bla*_NDM-13_ carried by a transmissible IncI1 plasmid may cause an increased risk of *bla*_NDM-13_ transmission. IS*1294* may be involved in the mobilization and dissemination of *bla*_NDM-13_.

## Data availability statement

The datasets presented in this study can be found in online repositories. The names of the repository/repositories and accession number(s) can be found below: https://www.ncbi.nlm.nih.gov/genbank/, CP092911-CP092914.

## Ethics statement

The studies involving human participant were reviewed and approved by the Ethics Committee of the First Affiliated Hospital of Xiamen University. The participant provided his written informed consent to participate in this study.

## Author contributions

HX and XL contributed to the conception and design of the study. HX and XM provided this strain. YH and SZ performed laboratory experiments. YH, XM, SZ, and LF analyzed the data. YH wrote the manuscript. XL revised the manuscript. All authors have read and approved the manuscript.

## Funding

This study was funded by the Youth Foundation of the National Natural Science Foundation of China (81902104), Basic and Applied Basic Research Foundation of Guangdong Province (2021A1515220153), Basic and Applied Basic Research Foundation of Guangdong Province Natural Science Foundation (2022A1515012481), and Joint Research Projects of Health and Education Commission of Fujian Province (2019-WJ-42).

## Acknowledgments

We thank Dr. Kai Zhou (Shenzhen Institute of Respiratory Diseases, The First Affiliated Hospital (Shenzhen People’s Hospital), Southern University of Science and Technology, Shenzhen, China) for his revision of the manuscript.

## Conflict of interest

The authors declare that the research was conducted in the absence of any commercial or financial relationships that could be construed as a potential conflict of interest.

## Publisher’s note

All claims expressed in this article are solely those of the authors and do not necessarily represent those of their affiliated organizations, or those of the publisher, the editors and the reviewers. Any product that may be evaluated in this article, or claim that may be made by its manufacturer, is not guaranteed or endorsed by the publisher.
