# Supplementary material for: Emergence of a Salmonella Rissen ST469 clinical isolate carrying bla NDM-13 in China
Source: Front Cell Infect Microbiol. 2022 Aug 8;12:936649. doi: 10.3389/fcimb.2022.936649 (PMC9393422; doi:10.3389/fcimb.2022.936649)
Supplement: Supplementary file 1 [file Table_1.docx]

Supplementary Material

# Supplementary Table

Table S1. Information of resistance genes detected in SR33

| **Gene** | **Location** | **Resistance** |
| --- | --- | --- |
| *bla*_NDM-13_ | IncI1 plasimd | Carbapenem |
| *ble*_MBL_ | IncI1 plasimd | Bleomycin |
| *bla*_TEM-1_ | Chromosome | Beta-lactam |
| *aadA1* | Chromosome | Streptomycin |
| *aadA2* | Chromosome | Streptomycin |
| *cmlA1* | Chromosome | Chloramphenicol |
| *dfrA12* | Chromosome | Trimethoprim |
| *sul3* | Chromosome | Sulfonamide |
| *tet(A)* | Chromosome | Tetracycline |
