# Supplementary material for: Emergence of a Salmonella Rissen ST469 clinical isolate carrying bla NDM-13 in China
Source: Front Cell Infect Microbiol. 2022 Aug 8;12:936649. doi: 10.3389/fcimb.2022.936649 (PMC9393422; doi:10.3389/fcimb.2022.936649)
Supplement: Supplementary file 2 [file Table_2.docx]

Supplementary Material

# Supplementary Table

Table S2. Information of *S*. Rissen ST469 isolates downloaded from EnteroBase.

| **Strain name** | **source** | **country** | **collection date** |
| --- | --- | --- | --- |
| 102* | human | China | 2020 |
| QLUY822 | poultry | China | 2011 |
| 0810/DIA/2020-7T | food | China | 2020 |
| 1099_DIA_2020_1T | food | China | 2020 |
| 1542_DIA_2020-1T | food | China | 2020 |
| 2406_DGP_2020-5T | food | China | 2020 |
| R17.4246 | human | China | 2007 |
| JST04 | NA | China | NA |
| 1396DGP2019(14T) | food | China | 2019 |
| 296DIA2019(1T) | food | China | 2019 |
| 1099/DIA/2020-1T | food | China | 2020 |
| 1755DGP2019 | food | China | 2019 |
| 0396_DIA_2021_1T | food | China | 2021 |
| 0371_DGP_2021_4T | food | China | 2021 |
| 2296/DIA/2021-4T | food | China | 2021 |
| 0854_DGP_2021-2T | food | China | 2021 |
| 0849_DGP_2021-1T | food | China | 2021 |
| 1145_DIA_2020-1T | food | China | 2020 |
| 0872/DGP/2021-2T | food | China | 2021 |
| 0873/DGP/2021-5T | food | China | 2021 |
| 706/SIS/2018-1T | food | China | 2018 |
| GJ0703-2 | human | China | 2015 |
| 444/DIA-2020-1T | food | China | 2020 |
| QLUY815 | poultry | China | 2011 |
| R17.5428 | human | China | 2017 |
| QLULG3 | poultry | China | 2010 |
| 2614DGP2019-1T | food | China | 2019 |
| 254DIA2020-4T | food | China | 2020 |
| 1226SIS2018-3T | food | China | 2018 |
| 1226-3 | food | China | 2018 |
| QLUY707 | food | China | 2006 |
| QLUY410 | poultry | China | 2010 |
| FAR0094 | environment | China | 2011 |
| QLULF8 | poultry | China | 2011 |
| QLULO4 | poultry | China | 2011 |
| QLULE6 | poultry | China | 2007 |

* indicates that the strain comes from previous studies in our laboratory. NA, not available.
